# Perception of skin cancer risk and sun protective practices in individuals with vitiligo: a prospective international cross-sectional survey

**DOI:** 10.1007/s00403-024-02942-0

**Published:** 2024-05-22

**Authors:** Sarah Gonzalez, Arielle Carolina Mora Hurtado, Nicole C. Syder, Jack Rodman, Nada Elbuluk

**Affiliations:** 1 Wayne State University School of Medicine, Detroit, MI USA; 2 University of Wisconsin School of Medicine and Public Health, Madison, WI USA; 3 Department of Dermatology, University of Southern California Keck School of Medicine, 830 Flower Street, Suite 100, Los Angeles, CA 90017 USA; 4 Southern California Clinical and Translational Sciences Institute (SC CTSI), Los Angeles, CA USA

**Keywords:** Vitiligo, Photoprotection, Skin cancer, Patient education

## Abstract

Many individuals with vitiligo are uncertain about their skin cancer risk, phototherapy risks, and recommended sun protective practices. This study examined the perceived skin cancer risk and sun protective practices among individuals living with vitiligo. A secondary objective was to understand where participants obtain this information. This was a prospective cross-sectional study. An online survey was distributed to vitiligo support group leaders globally who shared the survey with their members. Individuals over the age of 18 and with vitiligo were included. There were 209 survey respondents, the majority were between the ages 35–54 (45.5%, *n* = 95), female (70.8%, *n* = 148), White (66.0%, *n* = 138). Nearly half of respondents believed they were at increased risk of skin cancer because of their vitiligo (45.5%, *n* = 95) and nearly a quarter (22.5%, *n* = 47) believed that phototherapy increased their risk of skin cancer. Having vitiligo affected sun protective practices with less than a quarter (24.4%, *n* = 51) of respondents using sunscreen daily or often prior to their vitiligo diagnosis in comparison to the majority of respondents (60.3%, *n* = 126) using it after their vitiligo diagnosis. The three most common sources where patients obtained information were the internet and social media (46.4%, *n* = 97), vitiligo support groups (23.4%, *n* = 49), and dermatologists (20.6%, *n* = 43). Despite evidence indicating a decreased risk of skin cancer in individuals with vitiligo and supporting the safety of narrowband ultraviolet B phototherapy, many participants believed they were at an increased risk of skin cancer. Findings were sub-stratified and showed differences in sunscreen usage based on gender, skin color, and percent depigmentation. This study also found nearly half of respondents obtained information related to vitiligo from the internet and social media. The number of participants may limit the generalizability of the findings. Survey questionnaires are also subject to response bias. The findings from this study highlight demographic variations in sunscreen usage which may help guide the development of targeted interventions to improve sun protective behaviors among diverse populations with vitiligo. In addition, this study suggests certain sun protective practices and skin cancer risk perceptions may vary based on extent of depigmentation. Lastly, this study also demonstrates the internet and social media as a popular source for obtaining information, emphasizing the need for dermatologists to leverage various online communication channels to help disseminate accurate information.

**Supplementary Information:**

The online version contains supplementary material available at 10.1007/s00403-024-02942-0.

## Introduction

Vitiligo is an acquired, autoimmune, chronic disorder characterized by depigmentation of the skin, affecting approximately 0.5–2% of the global population [1, 2]. This depigmentation has often led to concerns that individuals with vitiligo may be at greater risk of sunburns and skin cancer. Multiple cohort studies have fortunately shown a lower risk of skin cancer in individuals with vitiligo, including nonmelanoma skin cancer (NMSC) and malignant melanoma (MM) compared to the general population [1–3]. Despite this data, many individuals with vitiligo remain uncertain about their risk of skin cancer and recommended sun protective practices. The primary objective of our study was to investigate the perceptions of skin cancer risk and sun protection practices among individuals with vitiligo. This study also aimed to identify the sources from which individuals with vitiligo acquire this information.

## Methods and materials

### Study design

This study was a prospective cross-sectional survey. Demographic information was collected. The self-reported questionnaire queried individuals about their course of vitiligo, history of skin cancer, sun protective practices, perception of overall skin cancer risk and perception of skin cancer risk related to phototherapy treatment. Additional questions inquired as to where they obtained information regarding their vitiligo diagnosis, risk for skin cancer, risks of phototherapy and recommended sun protective practices.

### Participant recruitment

Subjects were recruited through national and international support groups. These support groups were identified through the Global Vitiligo Foundation, My Vitiligo Team, and social media platforms (Facebook, Instagram, Twitter). A total of twenty-six support groups were contacted. The leaders of these support groups were contacted via email explaining the purpose of the study and requesting their support in disseminating the survey to their support group members. Upon agreement from the support group leaders, they were provided with the anonymous survey link to distribute to their support group members.

### Survey development

The survey questionnaire was developed using REDCap. The questionnaire consisted of 19 questions regarding participants’ course of vitiligo, history of skin cancer, perceived risk of skin cancer overall and in relation to phototherapy, sun protective practices, and sources of information about vitiligo.

### Inclusion criteria

Individuals aged 18 years and older with a diagnosis of vitiligo were eligible to participate in the survey. To ensure participant anonymity, no identifiable information was collected for this survey study.

### Data collection

Participants accessed the survey through an anonymous survey link provided to the support group leaders. The survey was completed online, allowing participants to respond to the questions at their convenience. All data collected were anonymous, with no personal identifiers being recorded.

### Statistical analysis

Summary statistics for demographics and survey questions were presented using frequency and percentages. Associations between categorical variables of interest were evaluated using appropriate statistical tests, such as Pearson’s chi-square or Fisher’s exact test. All statistical analyses were conducted using R version 4.2.1. A significance level of *p* < 0.05 was considered statistically significant.

### Ethical considerations

This study obtained ethical approval from the USC Institutional Review Board (IRB). Participant anonymity and confidentiality were strictly maintained throughout the survey process. The voluntary nature of participation was emphasized. No incentives for survey completion were offered.

## Results

Out of the twenty-six support groups contacted, six groups responded, with a total of 209 individuals participating in the survey (Table 1). The majority of the respondents were between the ages 35–54 (45.5%, *n* = 95), female (70.8%, *n* = 148), White (66.0%, *n* = 138) and with a college and/or graduate level education (74.7%; *n* = 156). A majority of respondents were non-skin of color (SOC) (66.0%, *n* = 138). The majority of respondents were from the United States and the international respondents were from the United Kingdom and Ghana.

**Table 1:** Demographic characteristics of survey respondents (*n* = 209)

Variable	Frequency (%)
Age	
18–34 years	48 (23.0%)
35–54 years	95 (45.5%)
55+ years	66 (31.6%)
Gender	
Male	59 (28.2%)
Female	148 (70.8%)
Nonbinary/nonconforming	1 (0.5%)
Prefer not to respond	1 (0.5%)
Transgender	0
Race/ethnicity	
White	138 (66.0%)
Asian	27 (12.9%)
Black/African American	22 (10.5%)
Hispanic/Latino	20 (9.6%)
Native Hawaiian/Pacific Islander	1 (0.5%)
Other	1 (0.5%)
American Indian/Alaska Native	0
Biracial/multiracial	0
Skin of color^a^	
Non-skin of color	138 (66.0%)
Skin of color	71 (34.0%)
Highest level of education	
Elementary	1 (0.5%)
Middle school	3 (14.4%)
High school	49 (23.4%)
Undergraduate	52 (24.9%)
Graduate	104 (49.8%)

^a^Skin of color included all non-white respondents in the study

The majority of respondents had been diagnosed with vitiligo by a dermatologist (84.2%, *n* = 176) and reported having vitiligo for 10 years or more (73.2%, *n* = 153). Percent depigmentation was self-reported to be less than 10% (23.9%, *n* = 50), 10–20% (27.3%, *n* = 57), 20–50% (23.9%, *n* = 50), and >50% (24.9%, *n* = 52).

While a majority of participants had no prior history of skin cancer (96.7%, *n* = 202), nearly half of respondents believed they were at increased risk of skin cancer because of their vitiligo (45.5%, *n* = 95). Respondents most often thought they were at an increased risk of melanoma (87.4%, *n* = 83), basal cell carcinoma (34.7%, *n* = 33), squamous cell carcinoma (29.5%, *n* = 28), and or all three skin cancers (24.2%, *n* = 23). The most commonly perceived factors felt to increase skin cancer risk included sun exposure (62.7%, *n* = 131) followed by having vitiligo (17.2%, *n* = 36), family history of skin cancer (9.6%, *n* = 20), diet (1.0%, *n* = 2) and other (9.6%, *n* = 20). Nearly a quarter of respondents (22.5%, *n* = 47) believed that phototherapy increased their risk of skin cancer and over two-thirds (67.5%, *n* = 141) did not know whether phototherapy increased their skin cancer risk.

Less than a quarter (24.4%, *n* = 51) of respondents reported using sunscreen daily or often *prior* to their vitiligo diagnosis, however *after* being diagnosed with vitiligo, the majority of respondents (60.3%, *n* = 126) reported using sunscreen daily or often because of their vitiligo. The majority of respondents also reported wearing ultraviolet protection factor (UPF) clothing when out in the sun (66.0%, *n* = 138). Of the 177 respondents who wore sunscreen, the majority (84.7%) reported using SPF (sun protection factor) 30 or higher (86.9%, *n* = 180), nearly a third wore it daily (31.3%, *n* = 55), close to half wore it often (43.8%, *n* = 77), and a quarter wore it rarely (25.0%, *n* = 44). Although the majority of individuals wore sunscreen, only two thirds (38.9%, *n* = 81) reported reapplying every 2–3 h when outside (61.1%, *n* = 127) (Tables 2, 3). Nearly half (43.1%, *n* = 90) of respondents reported their concern for skin cancer impacted their outdoor activities.

**Table 2:** Sunscreen use in relation to vitiligo diagnosis

Variable	Did you wear sunscreen daily or often *prior* to having vitiligo?		*p* value
	No (*n* = 158) Frequency (%)	Yes (*n* = 51) Frequency (%)	
*Age group*			0.755
18–34 years	36 (22.8%)	12 (23.5%)	
35–54 years	70 (44.3%)	25 (49.0%)	
55+ years	52 (32.9%)	14 (27.5%)	
*Gender*			0.004*
Male	53 (33.5%)	6 (11.8%)	
Female	103 (65.2%)	45 (88.2%)	
Nonbinary/nonconforming**	1 (0.6%)	0	
Prefer not to respond**	1 (0.6%)	0	
Transgender**	0	0	
*Race/ethnicity*			0.422
White	101 (63.9%)	37 (72.5%)	
Asian	21 (13.3%)	6 (11.8%)	
Black/African American	19 (12.0%)	3 (5.9%)	
Hispanic/Latino	16 (10.1%)	4 (7.8%)	
Native Hawaiian/Pacific Islander**	1 (0.6%)	0	
Other**	0	1 (2.0%)	
American Indian/Alaskan**	0	0	
Biracial/multiracial**	0	0	
*Skin of color*			0.258
Non-skin of color	101 (63.9%)	37 (72.5%)	
Skin of color	57 (36.1%)	14 (27.5%)	

Variable	Do you wear sunscreen daily or often because you have vitiligo?		*p* value
	No (*n* = 83) Frequency (%)	Yes (*n* = 126) Frequency (%)	
*Age group*			0.006*
18–34 years	21 (25.3%)	27 (21.4%)	
35–54 years	46 (55.4%)	49 (38.9%)	
55+ years	16 (19.3%)	50 (39.7%)	
*Gender*			0.007*
Male	32 (38.6%)	27 (21.4%)	
Female	50 (60.2%)	98 (77.8%)	
Nonbinary/nonconforming**	0	1 (0.8%)	
Prefer not to respond**	1 (1.2%)	0	
Transgender**	0	0	
*Race/ethnicity*			0.001*
White	42 (50.6%)	96 (76.2%)	
Asian	18 (21.7%)	9 (7.1%)	
Black/African American	13 (15.7%)	9 (7.1%)	
Hispanic/Latino	10 (12.1%)	10 (7.9%)	
Native Hawaiian/Pacific Islander**	0	1 (0.8%)	
Other	0	1 (0.8%)	
American Indian/Alaskan**	0	0	
Biracial/multiracial**	0	0	
*Skin of color*			<0.001*
Non-skin of color	42 (50.6%)	96 (76.2%)	
Skin of color	41 (49.4%)	30 (23.8%)	

Variable	Do you wear protective clothing (UPF clothing) when out in the sun?		*p* value
	No (*n* = 71) Frequency (%)	Yes (*n* = 138) Frequency (%)	
*Age group*			0.41
18–34 years	20 (28.2%)	28 (20.3%)	
35–54 years	29 (40.8%)	66 (47.8%)	
55+ years	22 (31.0%)	44 (31.9%)	
*Gender*			0.033*
Male	25 (35.2%)	34 (24.6%)	
Female	44 (62.0%)	104 (75.4%)	
Nonbinary/nonconforming**	1 (1.4%)	0	
Prefer not to respond**	1 (1.4%)	0	
Transgender**	0	0	
*Race/ethnicity*			0.032*
White	43 (60.6%)	95 (68.8%)	
Asian	7 (9.9%)	20 (14.5%)	
Black/African American	13 (18.3%)	9 (6.5%)	
Hispanic/Latino	6 (8.5%)	14 (10.1%)	
Native Hawaiian/Pacific Islander**	1 (1.4%)	0	
Other**	1 (1.4%)	0	
American Indian/Alaskan**	0	0	
Biracial/multiracial**	0	0	
*Skin of color*			0.232
Non-skin of color	43 (60.6%)	95 (68.8%)	
Skin of color	28 (39.4%)	43 (31.2%)	

Variable	Do you reapply sunscreen every 2–3 h when outside?		*p* value
	No (*n* = 127) Frequency (%)	Yes (*n* = 81) Frequency (%)	
*Age group*			0.196
18–34 years	34 (26.7%)	14 (17.3%)	
35–54 years	52 (40.9%)	42 (51.9%)	
55+ years	41 (32.3%)	25 (30.9%)	
*Gender*			0.037*
Male	43 (33.9%)	16 (19.8%)	
Female	82 (64.6%)	65 (80.3%)	
Nonbinary/nonconforming**	1 (0.8%)	0	
Prefer not to respond**	1 (0.8%)	0	
Transgender**	0	0	
*Race/ethnicity*			<0.001*
White	71 (55.9%)	66 (81.5%)	
Asian	23 (18.1%)	4 (4.9%)	
Black/African American	21 (16.5%)	1 (1.2%)	
Hispanic/Latino	10 (7.9%)	10 (12.4%)	
Native Hawaiian/Pacific Islander**	1 (0.8%)	0	
Other**	1 (0.8%)	0	
American Indian/Alaskan**	0	0	
Biracial/multiracial**	0	0	
*Skin of color*			<0.001*
Non-skin of color	71 (55.9%)	66 (81.5%)	
Skin of color	56 (44.1%)	15 (18.5%)	

Values were bolded to highlight the statistically significant *p*-values

Numbers represent frequency (column percent)

* Significant at *p* < 0.05 (Fisher’s exact)

** For any variable with less than 3 responses, the data was not included in the analysis between groups

**Table 3:** SPF level utilized by demographic group

Variable	What SPF level do you look for in sunscreen?			*p* value
	15+ (*n* = 27) Frequency (%)	30+ (*n* = 94) Frequency (%)	55+ (*n* = 86) Frequency (%)	
*Age group*				0.194
18–34 years	11 (40.7%)	20 (21.3%)	17 (19.8%)	
35–54 years	11 (40.7%)	42 (44.7%)	40 (46.5%)	
55+ years	5 (18.5%)	32 (34.0%)	29 (33.7%)	
*Gender*				0.003*
Male	14 (51.9%)	25 (26.6%)	18 (20.9%)	
Female	12 (44.4%)	68 (72.3%)	68 (79.1%)	
Nonbinary/nonconforming**	0	1 (1.1%)	0	
Prefer not to respond**	1 (3.7%)	0	0	
Transgender**	0	0	0	
*Race/ethnicity*				<0.001*
White	9 (33.3%)	69 (73.4%)	60 (69.8%)	
Asian	5 (18.5%)	14 (14.9%)	8 (9.3%)	
Black/African American	10 (37.0%)	6 (6.4%)	4 (4.7%)	
Hispanic/Latino	3 (11.1%)	5 (5.3%)	12 (14.0%)	
Native Hawaiian/Pacific Islander**	0	0	1 (1.2%)	
Other**	0	0	1 (1.2%)	
American Indian/Alaskan**	0	0	0	
Biracial/multiracial**	0	0	0	
*Race (skin of color vs. non)*				<0.001*
Non-skin of color	9 (33.3%)	69 (73.4%)	60 (69.8%)	
Skin of color	18 (66.7%)	25 (26.6%)	26 (30.2%)	

Numbers represent frequency (column percent)

* Significant at *p* < 0.05 (Fisher’s exact). These values are also bolded.

** For any variable with less than 3 responses, the data was not included in the analysis between groups

**Fig. 1:**
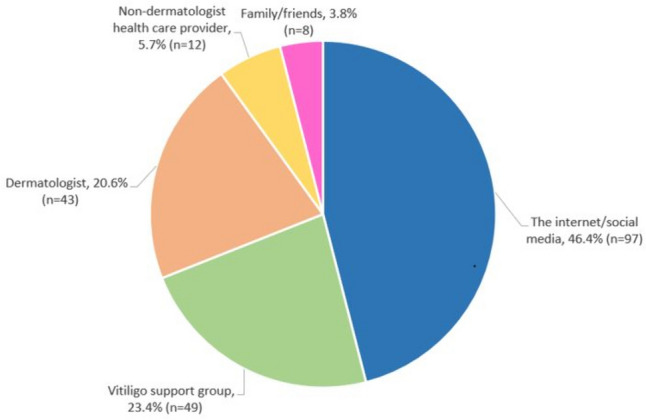
Information resources utilized by individuals with vitiligo

Regarding their preferred sources to obtain information about vitiligo and skin cancer risk, respondents most commonly relied on the internet and social media (46.4%, *n* = 97). This was followed by vitiligo support groups (23.4%, *n* = 49), their dermatologist (20.6%, *n* = 43), a non-dermatologist health care provider (5.7%, *n* = 12), and family or friends (3.8%, *n* = 8) (Fig. 1).

### Age sub-analyses

The survey data was further sub-stratified and analyzed by demographic groups including age, gender, and race/ethnicity. The 18–34 age group was categorized as the “young” cohort, the 35–54 age group as “middle aged,” and the 55+ age group as the “older” cohort. Middle aged (39.7%, *n* = 52) and older (38.9%, *n* = 51) cohorts were more likely to report sun exposure as the factor most affecting their skin cancer risk as compared to the young cohort (21.4%, *n* = 28, *p* = 0.035). Middle aged individuals (58.3%, *n* = 21) were more likely to report their vitiligo diagnosis most affecting their skin cancer risk when compared to the young (19.4% *n* = 7) and the older cohorts (22.2%, *n* = 8, *p* = 0.035). With regards to sun- protective practices, individuals from the older cohort (39.7%, *n* = 50) were more likely to wear sunscreen daily due to their vitiligo diagnosis compared to the young cohort (21.4%, *n* = 27, *p* = 0.006).

Moreover, in regard to preferred sources of information for vitiligo and skin cancer risk, individuals belonging to the older group (63.2%, *n* = 31) were more likely to utilize vitiligo support groups for this information as compared to the young (8.2%, *n* = 4) and middle aged cohorts (28.6%, *n* = 14, *p* < 0.001). This is in contrast to individuals from the middle aged cohort (53.6%, *n* = 52), who more often utilized the internet and social media to obtain this information compared to the young (22.7%, *n* = 22) and older cohorts (23.7%, *n* = 23, <0.001). The middle aged cohort (75%, *n* = 6) also more often reported obtaining vitiligo and skin cancer risk information from their family or friends as compared to the young and older cohorts. Regarding dermatologists as a source of information, the young (41.9%, *n* = 18) and middle aged (41.9%, *n* = 18) groups were more likely to obtain this formation from a dermatologist as compared to the older cohort (16.3%, *n* = 7, *p* < 0.001).

**Fig. 2:**
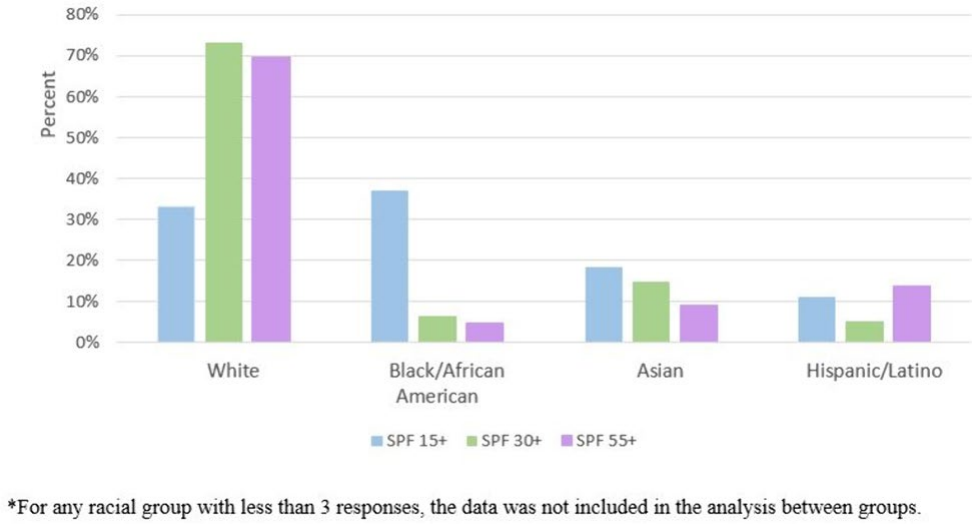
Racial/ethnic variation in SPF utilization

### Gender sub-analyses

Female participants were more likely to wear sunscreen daily than males (88.2%, *n* = 45 vs. 11.8%, *n* = 6, *p* = 0.004) prior to their vitiligo diagnosis and following their vitiligo diagnosis (77.8%, *n* = 98 vs. 21.4%, *n* = 27, *p* = 0.007). Women were also more likely to wear sun protective (UPF) clothing than men (75.4%, *n* = 104 vs. 24.6%, *n* = 34, *p* = 0.033). Female respondents showed a preference for sunscreens with SPF 30+ compared to males (72.3%, *n* = 68 vs. 26.6%, *n* = 25, *p* = 0.003). Female respondents also showed a higher preference for sunscreens with SPF 55+ compared to males (79.1%, *n* = 68 vs. 20.9%, *n* = 18, *p* = 0.003). Males tended to more often choose sunscreen with lower SPF of 15+ (51.9%, *n* = 14). Female participants also reported greater tendency to reapply sunscreen every 2–3 h when outdoors compared to males (80.3%, *n* = 65 vs. 19.8%, *n* = 16, *p* = 0.037).

### Race/ethnicity sub-analyses

SOC and race/ethnicity also showed differences in sun protective behaviors amongst those with vitiligo. Non-SOC individuals with vitiligo were more likely to wear sunscreen daily due to their vitiligo diagnosis compared to those with SOC (76.2%, *n* = 96 vs. 23.8%, *n* = 30, *p* < 0.001). Non-SOC participants also preferred sunscreen with SPF 30+ (73.4%, *n* = 69) and SPF 55+ (69.8%, *n* = 60), while those with SOC more often used sunscreen with SPF 15+ (66.7%, *n* = 18, *p* < 0.001). Furthermore, SOC individuals were less likely to reapply sunscreen every 2–3 h (18.5%, *n* = 15) compared to non-SOC (81.5%, *n* = 66; *p* < 0.001). White and Asian participants preferred sunscreens with higher SPF 30+ or 55+ (71.6%, *n* = 129 and 12.2%, *n* = 22 respectively) compared to Black respondents who showed a greater tendency to choose sunscreens with lower SPF 15+ (37%, *n* = 10, *p* < 0.001) (Fig. 2). White individuals (68.8%, *n* = 95) were more likely to report wearing sun protective UPF clothing when out in the sun compared to Black respondents (6.5%, *n* = 9, *p* = 0.032).

Regarding perceived increased risk of skin cancer, non-SOC (77.9%, *n* = 74) respondents more often reported believing they were at increased of skin cancer due to their vitiligo when compared to those with SOC (22.1%, *n* = 21, *p* = 0.001). Individuals with non-SOC (74.8%, *n* = 98) reported sun exposure as most contributing to their skin cancer risk when compared to SOC respondents (25.2%, *n* = 33, *p* = 0.003). In contrast, SOC respondents (60%, (*n* = 12) were more likely to report “other” factors as most contributing to their skin cancer risk when compared to non-SOC individuals (40%, *n* = 8, *p* = 0.003). There were no differences in perceived risk of phototherapy and skin cancer when data was analyzed by demographic groups.

### Disease severity sub-analyses

Sun protective practices differed amongst individuals with greater extent of depigmentation compared to those with more localized vitiligo (Table 4). Among respondents with >50% depigmentation, 82.7% (*n* = 43) reported using sunscreen daily or often due to their vitiligo diagnosis, compared to 48.0% (*n* = 24) of individuals with <10% depigmentation (*p* = 0.001). Individuals with greater extent of depigmentation were also more likely to prefer SPF 55+ sunscreen compared to those with less extensive vitiligo, who showed a greater tendency to choose sunscreens with lower SPF (*p* = 0.010). In regard to sunscreen reapplication, 59.6% (*n* = 31) of individuals with >50% depigmentation reported reapplying sunscreen every 2–3 h when outside, as compared to 22.0% (*n* = 11) of individuals with <10% depigmentation (*p* = 0.001). Furthermore, individuals with a greater percentage of depigmentation more frequently reported wearing UPF clothing compared to those with more localized vitiligo (*p* = 0.002). With respect to skin cancer risk perceptions, the majority of respondents (63.5%, *n* = 33) with >50% depigmentation reported their concern for skin cancer impacted their outdoor activities, as compared to only a third of individuals (30.0%, *n* = 15) with <10% depigmentation reporting this concern (*p* = 0.003).

**Table 4:** Skin cancer risk perceptions and sun protective practices by percent of depigmentation

Variable	Percent (%) of depigmentation				*p* value
	<10% (*n* = 50)	10–20% (*n* = 57)	20–50% (*n* = 50)	>50% (*n* = 52)	
Do you wear sunscreen daily or often because you have Vitiligo?					0.001*
No	26 (52.0%)	25 (43.9%)	23 (46.0%)	9 (17.3%)	
Yes	24 (48.0%)	32 (56.1%)	27 (54.0%)	43 (82.7%)	
Do you wear protective clothing (UPF clothing) when out in the sun?					0.002*
No	27 (54.0%)	19 (33.3%)	15 (30.0%)	10 (19.2%)	
Yes	23 (46.0%)	38 (66.7%)	35 (70.0%)	42 (80.8%)	
What SPF level do you look for in sunscreen?					0.010*
15+	8 (16.0%)	11 (19.3%)	7 (14.6%)	1 (1.9%)	
30+	28 (56.0%)	25 (43.9%)	22 (45.8%)	19 (36.5%)	
55+	14 (28.0%)	21 (36.8%)	19 (39.6%)	32 (61.5%)	
NA/missing	0	0	2	0	
Do you reapply sunscreen every 2–3 h when outside?					0.001*
No	39 (78.0%)	37 (66.1%)	30 (60.0%)	21 (40.4%)	
Yes	11 (22.0%)	19 (33.9%)	20 (40.0%)	31 (59.6%)	
NA/missing	0	1	0	0	
Does your concern for skin cancer impact your outdoor activities?					0.003*
No	35 (70.0%)	32 (56.1%)	33 (66.0%)	19 (36.5%)	
Yes	15 (30.0%)	25 (43.9%)	17 (34.0%)	33 (63.5%)	

Values were bolded to highlight the statistically significant *p*-values

Numbers represent frequency (column percent)

* Significant at *p* < 0.05

## Discussion

The depigmentation caused by vitiligo can raise concern about susceptibility to sunburns and skin cancer risk among individuals with vitiligo. Fortunately, several studies have consistently shown a paradoxical inverse association between vitiligo and skin cancer, including NMSC and MM [1–3]. Paradisi et al. [3] conducted a nonconcurrent cohort study involving 10,040 Italian patients with vitiligo and observed a markedly reduced incidence of melanoma and NMSC in this population. A study by Teulings et al. [2] surveyed 1307 vitiligo patients and their partners, reporting a significantly decreased risk of MM and NMSC among Dutch individuals with vitiligo. This inverse relationship is further exemplified by melanoma-associated depigmentation, which is a positive prognostic indicator in MM patients, leading to significantly improved 5-year survival rates [4].

Most recently, a United Kingdom cohort study involving 15,156 vitiligo cases matched to 60,615 controls, demonstrated a 38% decreased risk of new-onset skin cancer, including both NMSC and MM subtypes, in individuals with vitiligo compared to the general population controls [5]. Despite the existing data suggesting a lower risk of skin cancer among individuals with vitiligo, many individuals with vitiligo perceive they are at increased risk of skin cancer due to their vitiligo diagnosis [6]. A cross sectional study regarding sun protective habits in patients with vitiligo demonstrated that nearly half of the participants believed they were at increased risk of skin cancer because of their vitiligo [6]. Similarly, in our survey almost half of respondents reported believing they were at increased risk for skin cancer due to their vitiligo. These gaps in perception and knowledge amongst those with vitiligo highlights a need for increased awareness and patient education.

Having a diagnosis of vitiligo also seems to affect individuals’ sun protective practices. In our survey, while only 24.4% of respondents reporting wearing sunscreen daily or often *prior* to their vitiligo diagnosis, *after* being diagnosed with vitiligo this increased to 60.3% of respondents wearing sunscreen daily or often. We observed differences in sun protective practices between genders and amongst racial/ethnic groups. Female respondents consistently displayed safer sun protective practices, including before and after their vitiligo diagnosis, reapplying sunscreen more frequently, and preferring higher SPF levels than male respondents. This trend was also observed with our non-SOC compared to our SOC respondents.

A cross sectional study investigating sun protective behaviors among individuals with vitiligo demonstrated higher rates of high-SPF sunscreen use among vitiligo patients compared to the general population [6]. A retrospective data analysis in Australia found that individuals with vitiligo had better sun protective behaviors compared to the general Australian population [7]. A cross-sectional study conducted by Bhatia et al. [8] demonstrated that nearly half of all participants believed they were at increased risk of skin cancer due to their vitiligo. Interestingly the same study found that individuals in a vitiligo support group were less likely to believe they were at increased risk of skin cancer due to their vitiligo and exhibited safer sun behavior, including reapplying sunscreen more frequently, avoiding peak UV exposure, and had decreased sunburns with skin peeling compared to those who did not belong to a support group [8].

Additionally, phototherapy was also perceived by nearly a quarter of respondents (22.5%) to increase skin cancer risk despite data showing that it, particularly NB UVB phototherapy, is a common and safe treatment for vitiligo and does not increase risk for skin cancer [9, 10]. Just over two-thirds of survey respondents did not know if phototherapy affected their risk of skin cancer. Multiple studies have supported the safety of phototherapy including in the treatment of vitiligo. Kim et al. [11] did not find an increased risk of MM or NMSC in individuals with vitiligo after receiving phototherapy. Another retrospective 10-year cohort study found that prolonged NBUVB phototherapy did not lead to a higher risk of NSMC or MM in patients with vitiligo [10]. In a meta-analysis, Wu et al. [12] found no significant association between NBUVB phototherapy and risk of MM and NMSC in patients with vitiligo. The authors also noted the risk was not affected by the number of phototherapy sessions and no significant difference was observed in skin cancer risk in patients from Europe compared to those from East Asia [12].

In addition to investigating knowledge, beliefs, and perceptions of individuals with vitiligo, this study also uniquely investigated where individuals with vitiligo obtain their health-related information. Nearly half of our respondents obtained information related to vitiligo from the internet and social media. Vitiligo support groups and dermatologists were the next two most common resources. Our study also demonstrated differences across age groups with middle aged adults most often utilizing the internet and social media as compared to older adults, who were more likely to utilize vitiligo support groups. This highlights the positive and educational role that dermatologists can play in utilizing online and support group platforms to effectively disseminate correct information to individuals with vitiligo.

This study also offered new insights into demographic differences in the vitiligo community with regards to their vitiligo related knowledge and practices. This study demonstrated a variation in compliance with sun protective measures based on percentage of depigmentation. A majority of individuals with >50% depigmentation reported reapplying sunscreen, wearing UPF clothing, and selecting sunscreens with higher SPF levels. In addition, skin cancer risk perceptions also varied based on extent of vitiligo. Over two-thirds of individuals with >50% depigmentation reported their concern for skin cancer impacted their outdoor activities, in contrast to only a third of individuals with <10% depigmentation reporting this concern. Furthermore, our study also revealed a variation in sunscreen usage based on gender and racial/ethnic groups, with a majority of those who reported using sunscreen regularly, at higher SPF levels and reapplying sunscreen being White and female. These findings suggest the presence of potential sociocultural factors influencing sunscreen usage among gender and racial/ethnic groups. Further research is warranted to explore these factors and develop targeted interventions to improve sun protective behaviors among diverse populations with vitiligo.

Limitations of this study include the total number of participants which may limit the generalizability of the findings to the broader population of individuals with vitiligo. Additionally, the survey relied on self-reported data, which is subject to response bias and may not always accurately reflect actual behavior of all individuals with vitiligo. Future studies exploring skin cancer perceptions and sun protective behaviors in individuals with vitiligo should further investigate how these perceptions and behaviors may relate to the distribution and involvement of photo-exposed areas as well as prior phototherapy treatment.

In conclusion, this study provides valuable insights into the knowledge, beliefs, and perceptions of skin cancer risk, phototherapy safety, and sun protective practices among those living with vitiligo. Despite evidence indicating a decreased risk of skin cancer in individuals with vitiligo and supporting the safety of NBUVB phototherapy for vitiligo, many participants felt they were at increased risk of skin cancer and thought phototherapy increased this risk or were unsure of the risks of phototherapy. Sun protective practices were affected by having the diagnosis of vitiligo and were further differentiated by demographic variables such as gender and race/ethnicity. Furthermore, this study provides additional knowledge about where individuals with vitiligo obtain their knowledge in relation to their vitiligo. This study emphasizes the need for dermatologists and dermatology organizations to use various communication channels, including social media and the internet, patient visits and support groups to help disseminate accurate information regarding skin cancer risk with vitiligo and phototherapy as well recommendations for sun protective behaviors across demographic groups.

### Supplementary Information

Below is the link to the electronic supplementary material.

Supplementary file1 (PDF 259 KB)

## Data Availability

No datasets were generated or analysed during the current study.

## Abbreviations

MM: Malignant melanoma
NBUVB: Narrowband ultraviolet B
NMSC: Nonmelanoma skin cancer
SOC: Skin of color
SPF: Sun protection factor
UPF: Ultraviolet protective factor

## References

[1] Rodrigues M (2017). Skin cancer risk (nonmelanoma skin cancers/melanoma) in vitiligo patients. Dermatol Clin.

[2] Teulings HE, Overkamp M, Ceylan E (2013). Decreased risk of melanoma and nonmelanoma skin cancer in patients with vitiligo: a survey among 1307 patients and their partners. Br J Dermatol.

[3] Paradisi A, Tabolli S, Didona B, Sobrino L, Russo N, Abeni D (2014). Markedly reduced incidence of melanoma and nonmelanoma skin cancer in a nonconcurrent cohort of 10,040 patients with vitiligo. J Am Acad Dermatol.

[4] Naveh HP, Rao UN, Butterfield LH (2013). Melanoma-associated leukoderma—immunology in black and white?. Pigment Cell Melanoma Res.

[5] Ferguson J, Eleftheriadou V, Nesnas J (2023). Risk of melanoma and nonmelanoma skin cancer in people with vitiligo: United Kingdom population-based cohort study. J Invest Dermatol.

[6] Selçuk LB, Katkat E, Arıca DA, Yaylı S, Bahadır S (2020). Sun-protection habits and knowledge of patients with vitiligo. Acta Dermatovenerol Alp Pannonica Adriat.

[7] McDonald PB, Zapata L, Rodrigues M (2018). Sunscreen habits and skin cancer rates in patients with vitiligo in Australia. Australas J Dermatol.

[8] Bhatia B, Kechichian E, Eleftheriadou V (2019). Habits and risk perception associated with sun exposure in vitiligo patients according to their participation in a patients’ organization. J Eur Acad Dermatol Venereol.

[9] Rodrigues M, Ezzedine K, Hamzavi I, Pandya AG, Harris JE, Vitiligo WG (2017). New discoveries in the pathogenesis and classification of vitiligo. J Am Acad Dermatol.

[10] Bae JM, Ju HJ, Lee RW (2020). Evaluation for skin cancer and precancer in patients with vitiligo treated with long-term narrowband UV-B phototherapy. JAMA Dermatol.

[11] Kim HS, Kim HJ, Hong ES (2020). The incidence and survival of melanoma and nonmelanoma skin cancer in patients with vitiligo: a nationwide population-based matched cohort study in Korea. Br J Dermatol.

[12] Wu YH, Chou CL, Chang HC (2022). Risk of skin cancer after ultraviolet phototherapy in patients with vitiligo: a systematic review and meta-analysis. Clin Exp Dermatol.

