# Supplementary material for: Perception of skin cancer risk and sun protective practices in individuals with vitiligo: a prospective international cross-sectional survey
Source: Arch Dermatol Res. 2024 May 22;316(5):189. doi: 10.1007/s00403-024-02942-0 (PMC11111492; doi:10.1007/s00403-024-02942-0)
Supplement: Supplementary file 1 — Supplementary file1 (PDF 259 KB) [file 403_2024_2942_MOESM1_ESM.pdf]

Vitiligo and Skin Cancer Survey Questions

1. Age Range
   - A) 18-24
   - B) 25-34
   - C) 35-44
   - D) 45-54
   - E) 55-64
   - F) 65 and older

2. Gender
   - A) Male
   - B) Female
   - C) Transgender
   - C) Non-binary/Non-conforming
   - D) Prefer not to respond

3. Race/Ethnicity
   - A) American Indian or Alaska Native
   - B) Asian
   - C) Black or African American
   - D) Hispanic or Latino
   - E) Native Hawaiian or Other Pacific Islander
   - F) White

4. Highest level of education
   - A) Elementary
   - B) Middle School
   - C) Highschool
   - D) Undergraduate
   - E) Graduate

5. Were you diagnosed with vitiligo by a dermatologist?
   - A) Yes
   - B) No

5. Years with vitiligo diagnosis
   - A) <5 years
   - B) 5-10 years
   - C) 10-20 years

D) >20 years

7. % of depigmentation (areas with vitiligo)
A) <10 %
B) 10-20%
C) 20-50%
D) >50%

8. Do you use sunscreen?
A) Yes
B) No
If so, how often?
A) Daily
B)Often
C) Rarely

9. Do you wear sunscreen daily or often because you have vitiligo?
A) Yes
B) No
10. Did you wear sunscreen daily or often prior to having vitiligo?
A) Yes
B) No

11. Do you wear protective clothing (ie hats, UPF clothing) when out in the sun?
A) Yes
B) No

12. What SPF level do you look for in sunscreen?
A) 15+
B) 30+
C) 55+

13. Do you reapply your sunscreen every 2-3 hours when outside?
A) Yes
B) No

14. Where do you get most of your information regarding your vitiligo and risk for skin cancer from?
A) A health care provider
B) Dermatologist

C) The internet and social media
D) Family or friends
E) Vitiligo support group

15. Do you think you have an increased risk of skin cancer because of your vitiligo?
A) Yes
B) No

If yes, which type of skin cancer do you believe you are at an increased risk for?
A) Squamous Cell Cancer
B) Basal Cell Cancer
C) Melanoma
D) All of the above

16. Do you believe that phototherapy (light or laser therapy) for vitiligo affects skin cancer risk?
A) Yes
B) No
C) I don't know

17. Do you have any known history of skin cancer (ie basal cell, squamous cell, or melanoma)?
A) Yes
B) No

18. What factor do you feel most affects your risk of skin cancer?
A) Family history
B) Sun exposure
C) Diet
D) Vitiligo
E) Other

19. Does your concern for skin cancer impact your outdoor activities?
A) Yes
B) No
